# Evaluation of the Toxicity of Chemical and Biogenic Insecticides to Three Outbreaking Insects in Desert Steppes of Northern China

**DOI:** 10.3390/toxins14080546

**Published:** 2022-08-10

**Authors:** Wenbing Zhang, Hao Ren, Feilong Sun, Tingting Shen, Shuai Yuan, Xiwu Gao, Yao Tan

**Affiliations:** 1 College of Horticulture and Plant Protection, Inner Mongolia Agricultural University, Hohhot 010011, China; 2 College of Grassland, Resources and Environment, Inner Mongolia Agricultural University, Hohhot 010010, China; 3 Key Laboratory of Grassland Resources, Ministry of Education, Hohhot 010010, China; 4 Department of Entomology, China Agricultural University, Beijing 100193, China

**Keywords:** insecticide toxicity, leaf beetle, locust/grasshopper, bioassay, pest management

## Abstract

The locusts *Oedales asiaticus* (Bey-Bienko) and *Myrmeleotettix palpalis* (Zubovski) (Orthoptera Acrididae) and the leaf beetle *Galeruca daurica* (Joannis) (Coleoptera, Chrysomelidae) are economically devastating insect species in the desert steppes of Northern China. Control is mainly and frequently dependent on highly toxic chemicals. To date, there have been no complete and comprehensive reports of insecticide applications to these key pests. In this study, laboratory bioassays were carried out to determine and compare the toxicity of twelve insecticides to three outbreaking insects, *O. asiaticus*, *M. palpalis*, and *G. daurica,* from three typical desert steppe regions, SZWQ, XHQ and WLTQQ, respectively. The responses of the two locust species and the leaf beetle were evaluated by topical application and leaf dip bioassay techniques across a range of concentrations to develop dosage–mortality regressions. The insecticides tested included six chemical insecticides (β-cypermethrin, imidacloprid, phoxim, λ-cyhalothrin, methomyl, chlorantraniliprole) and six biogenic insecticides (spinosad, avermectin, rotenone, matrine, azadiracthin, and methoxyfenozide). The results showed that phoxim, λ-cyhalothrin, β-cypermethrin and spinosad showed highly toxic activity to *O.asiaticus*, *M. palpalis*, and *G. daurica*, while methonyl, chlorantraniliprole, and rotenone were moderately toxic to both locust species and the leaf beetle. The LC_50_ values of matrine, azadiractin, and avermectin were more than 1 μg a.i./adult for *O. asiaticus* and *M. palpalis*, the LC_50_ values of which were higher 2 g/L for *G. daurica*. Our findings complement information from previous similar studies and will inform future studies relating to the control of outbreaking insects, such as *O.asiaticus*, *M. palpalis*, and *G. daurica* in desert steppes of northern China. This study is also expected to provide basic data on the use of chemical and biogenic insecticides for application in desert steppes.

## 1. Introduction

In China, steppes account for 80% of vegetation and have high biodiversity and conservation value [1]. The northern steppes, accounting for 25% of the national grassland area, play important roles in protecting the ecological environment, developing the socio-economic growth of the regions, and conserving species diversity. However, a large area of steppes is being gradually degraded into desert due to increasing human activity [2]. Grass production has decreased by 30–50% since the 1950s and steppes have been turned into barren land and desert in some severely degraded areas [3]. Along with the reduced productivity of this grassland, ecosystem functions were significantly impaired, including vegetation cover and composition, species richness and diversity, the proportion of fine forage, aboveground and underground biomass, and carbon and nitrogen storage [4,5]. Desert steppes are the most arid grassland ecosystem type, occurring in areas with annual precipitation between 150 and 250 mm and under the influence of continental climatic conditions [6]. The *Allium* plant, *Allium polyrhizum* Turcz. ex Regel and several *Stipa* species are widely distributed in the typical desert steppe [7]. Desert steppe systems are generally sensitive to any external disturbance because of their fragile eco-environment system [8]. In recent decades, it is well known that mass outbreaks of pests have had a serious impact on desert steppes, and these outbreaks have been the subject of considerable research emphasis in northern China because of their impact on desert steppe ecosystem processes and functioning.

Sudden and large-scale pest occurrences in desert steppes of northern China have been monitored continuously for many years [9,10]. Outbreaks of these pests have had disastrous consequences for the grassland ecology and the local economy as a whole, with strongly negative effects on agriculture and the development of animal husbandry. The outbreak frequency and impact of the most commonly occurring pest species have been studied with the locusts *Oedaleus asiaticus* Bey-Bienko and *Myrmeleotettix palpalis* Zubovski (Orthoptera, Acrididae), and leaf beetle *Galeruca daurica* (Joannis) (Coleoptera, Chrysomelidae) reported as the three dominant insect species in the vast desert steppe areas of northern China [10,11,12,13].

The locust *Oedaleus asiaticus* (Orthoptera, Acrididae) is an important grass-feeding insect widely distributed in steppe and adjacent farmland throughout Northern Asian regions such as Russia, Mongolia, and China. It is reported to be particularly abundant in the Inner Mongolia steppe and has caused huge damage to the livestock industry and ecological environment for many years [14,15]. As the primary consumer, *O. asiaticus* has specific food preferences, and this extreme adaptation has dramatically reduced grassland productivity and competed with other native herbivores and farmed animals for food resources [16,17]. The other economically important insect species in farming-pastoral ecotones in arid areas, *M. palpalis*, has also caused great losses to the farming and crop planting industry and serious damage to steppes, impeding the development of animal husbandry [18]. Earlier studies have reported the locust species involved, their regional distribution [19], biological characteristics [20,21], feeding habits [22], trophic niches and resource utilization [23,24]. The two locust species occupy the same spatial niches and prefer short grass steppes and xerophytic habitats. They have similar feeding habits, mainly on plants of Poaceae and Compositae. The leaf beetle, *G. daurica,* feeds exclusively on *Allium* species (Liliaceae) in the desert steppes of the Inner Mongolia grasslands in China [25]. Sudden outbreaks of this pest were initially reported to have caused great losses to pasture in 2009 and damage has increased extensively year by year [26,27,28]. These three key insects have one generation annually and over winter lay eggs, which hatch in spring when the pastures turn green and grow.

The application of chemical insecticides with high efficiency, high selectivity, low toxicity and low residue to control insects in desert steppes has the advantages of a good insecticidal effect with rapid results, and is environmentally friendly [29,30]. At present, it is still necessary and effective to control insects when large-area and high-density outbreaks occur on the steppes [31], so chemical control agents should be chosen scientifically and used rationally according to the actual situation of pest density, species, local vegetation, and environmental conditions to maximize the advantages of chemical control and minimize its side effects [32]. Currently, the use of conventional insecticides (organophosphates, carbamates, pyrethroids, and neonicotinoids) remains the most commonly used control tactic in this grassland ecosystem [15,33].

The insecticides used to control grassland insects in this region are often applied as foliar application from the air [34], but it has been recognized that the misuse of chemicals was seriously disrupting the ecosystem’s function, polluting the environment, and inducing pest resurgence and resistance [35]. Highly effective and environmentally safe control approaches have been explored, and new measures and techniques have been put into use for pest management [36]. When compared with chemical insecticides, biogenic insecticides (such as microsporidia microbial metabolites, avermectin, and spinosad) and biological insecticides (such as azadirachtin and matrine) are a better control strategy because they have strong selectivity and are safe for humans and livestock.

Outbreaks of *O. asiaticus*, *M. palpalis* and *G. daurica* have caused remarkable grass yield losses and desert steppe deterioration in recent years, thereby putting tremendous pressures on this fragile ecosystem. According to the latest statistics from the China Pesticide Information Official Network, there are 35 pesticide products registered by the Ministry of Agriculture and Rural Affairs of China for grass locust control in China. Among them, the seven main chemical insecticide ingredients are dichlorvos, malathion, imidacloprid, β-cypermethrin, cypermethrin, avermectin and triazophos, and two biogenic insecticides of plant origin are matrine and azadirachtin. More registered insecticides are compounded and although many broad-spectrum insecticides with different modes of action were applied against the main insect species in this region, few studies about current insecticide toxicities and the resistances of the grass pests have been reported. The study reported here documented the susceptibilities of these three key pest species to six chemical insecticides and six bio-insecticides from 2021 to 2022 using both leaf-dip and topical applications in three typical desert steppe sites in northern China, which provides a theoretical basis for pest management strategies for steppe and grassland protection.

## 2. Results

### 2.1. Toxicity of Insecticides to Oedaleus asiaticus from the Three Regions Studied

*O. asiaticus* adults from SZWQ were most susceptible to the traditionally-used insecticides β-cypermethrin, imidacloprid, phoxim, and λ-cyhalothrin. The LC_50_ values of the eleven insecticides ranged from 213.48 ng a.i./adult (β-cypermethrin) to 11.99 μg a.i./adult (avermectin), a 56.16-fold range, compared with a much larger range in LC_50_ values for the other two regions. The toxicities of the eleven tested insecticides against *O. asiaticus* SZWQ populations, ranked from high to low (based on LC_50_ values), are β-cypermethrin > imidacloprid > phoxim > λ-cyhalothrin > spinosad > rotenone > methomyl > chlorantraniliprole > matrine > azadirachtin > avermectin. However, it was found that *O. asiaticus* collected from SZWQ was less susceptible to one chemical insecticide, chlorantraniliprole, as well as three biogenic insecticides, matrine, azadirachtin, and avermectin (Table 1).

The large range of toxicities of all the tested insecticides (avermectin LC_50_/β-cypermethrin LC_50_ = 52.69) to *O. asiaticus* adults from XHQ was remarkable, along with the toxicity range of several biogenic insecticides (avermectin LC_50_/spinosad LC_50_ = 26.41), although the ranking of the eleven tested insecticides from high to low toxicity was similar with that observed with *O. asiaticus* adults from SZWQ. For the *O. asiaticus* population collected from XHQ, the toxicity order was β-cypermethrin > phoxim > spinosad > imidacloprid > λ-cyhalothrin > rotenone > methomyl > chlorantraniliprole > matrine > azadirachtin > avermectin. Among the biogenic insecticides, avermectin was far less toxic than any of the other biocides, and the assay time was the longest (72 h). Based on LC_50_ values, *O. asiaticus* adults from XHQ were 1.17 and 1.24 times less susceptible to spinosad and rotenone, respectively, when compared with *O. asiaticus* adults from SZWQ, and relative toxicity indexes were 0.67 and 0.66 against matrine and azadirachtin.

The ranking of insecticide toxicity from high to low for the *O. asiaticus* adults collected from WLTQQ in 2022 followed a similar general pattern to the populations from SZWQ and XHQ, which was β-cypermethrin > phoxim > chlorantraniliprole > imidacloprid > λ-cyhalothrin > spinosad > rotenone > methomyl > azadirachtin > matrine. β-cypermethrin had the highest toxicity towards all *O. asiaticus* populations from SZWQ, XHQ, and WLTQQ when compared to the other test insecticides, indicating that it still retains good insecticidal activity in pest management in desert steppes. There was also a significant difference for chlorantraniliprole toxicity towards *O. asiaticus* between the WLTQQ, SZWQ, and XHQ populations (Table 1) while all the other biogenic insecticides except for spinosad showed low toxicity to *O. asiaticus* adults from the three regions.

### 2.2. Toxicity of Insecticides to Myrmeleotettix palpalis from SZWQ and XHQ

The large range of toxicities of all tested insecticides to *M. palpalis* populations from SZWQ and XHQ (avermectin LC_50_/spinosad LC_50_ = 61.21 for SZWQ and 60.64 for XHQ) was remarkable (Table 2). LC_50_ values show that both *M. palpalis* populations were most susceptible to spinosad followed by λ-cyhalothrin, imidacloprid and methomyl. *M. palpalis* from these two regions was the least susceptible to avermectin, usually followed by matrine in the SZWQ population, but followed by azadirachtin in the XHQ population. Rotenone and chlorantraniliprole also generally ranked low in toxicity among the eleven insecticides in *M. palpalis* field populations. The high toxicity of spinosad to SZWQ and XHQ populations indicated that a novel biogenic insecticide not used in this region showed highly toxic activity to *M. palpalis.* Meanwhile, *M. palpalis* from SZWQ and XHQ was relatively susceptible to λ-cyhalothrin (LC_50_ values were 70.73 ng a.i./adult and 88.39 ng a.i./adult) and β-cypermethrin (LC_50_ values were 279.73 ng a.i./adult and 215.54 ng a.i./adult), which demonstrated that pyrethroid insecticides generally have a strong application value for *M. palpalis* control.

### 2.3. Toxicity of Insecticides to Galeruca daurica from SZWQ and XHQ

*G. daurica* from SZWQ and XHQ were the most susceptible to imidacloprid as the LC_50_ values were 0.17 and 0.16 mg/L, respectively. The eleven insecticides can be ranked from high to low toxicity to *G. daurica* from SZWQ (based on LC_50_ values) in the order imidacloprid > λ-cyhalothrin > β-cypermethrin > phoxim > methomyl > spinosad > chlorantraniliprole > rotenone > matrine > methoxyfenozide > azadirachtin with overlaps of 95% fiducial limits (Table 3). Ranking insecticide toxicity from high to low for *G. daurica* from XHQ followed the similar general pattern as with *G. daurica* from SZWQ (imidacloprid > λ-cyhalothrin > phoxim > β-cypermethrin > spinosad > methomyl > chlorantraniliprole > rotenone > matrine > azadirachtin > methoxyfenozide). LC_50_ values for *G. daurica* from SZWQ and XHQ were 20,335, 18,641,and 18,856, 23,767 times more susceptible to imidacloprid, respectively, when compared with azadirachtin and methoxyfenozide. The high toxicity of imidacloprid to *G. daurica* from SZWQ and XHQ indicated that this neonicotinoid had good application values. Meanwhile, *G. daurica* from SZWQ and XHQ was more susceptible to λ-cyhalothrin (LC_50_ values were 0.73 and 0.56 mg/L) and β-cypermethrin (LC_50_ values were 1.57 mg/L and 1.06 mg/L). Both the SZWQ and XHQ *G. daurica* populations were least susceptible to azadirachtin and methoxyfenozide, which was usually followed by matrine. Rotenone also generally had low toxicity among the eleven insecticides while all the biogenic insecticides except spinosad showed high LC_50_ values for *G. daurica* from SZWQ and XHQ.

## 3. Discussion

In recent years, outbreaks of *O. asiaticus, M. palpalis,* and *G. daurica* have become frequent in the northern China steppes due to livestock overgrazing, extreme climate change, and consequent land desertification [33,37]. These pest species prefer to live on heavily-grazed steppes and mainly feed on gramineaceous and liliaceous plants such as stipa, wild rye, and allium [16,27,28]. The outbreaks have brought remarkable grass yield losses and led to grassland deterioration, thereby adversely affecting animal husbandry production and the development of the grassland industry. Chemical control is thus far the major means of controlling the outbreaks of insects in northern China [15,38]. Over the years, pyrethroids have been commonly used to control insect outbreaks in northern grasslands and steppes, among which β-cypermethrin was sprayed by local grassland stations as required, and used intensively on an annual basis. However, their effectiveness has been reported to have gradually diminished (based on insecticide resistance investigations), most likely because some insect populations in different regions have already developed resistance to these pyrethroids [16,39].

The toxicity evaluation showed that the insecticides β-cypermethrin, imidacloprid, phoxim, and λ-cyhalothrin displayed high toxicity to *O. asiaticus* by direct contact at the dosage recommended on the desert steppes from the three tested regions. There were great differences in the susceptibilities of *O. asiaticus* to the various insecticides, mainly due to the different mechanisms of action of each class of insecticide. The chemical insecticides (i.e., pyrethroids, organophosphates, and neonicotinoids) were more efficient in controlling *O. asiaticus* than the biogenic insecticides (i.e., avermectin, azadirachtin, and matrine) based on the bioassay results under laboratory conditions. Pyrethroids and organophosphates have a strong thixotropic and stomachic effect, and have shown greater efficiency than other insecticides tested when used by topical application, and which may be the consequence of their greater solubility in acetone (>450 g L^−1^ at 20 °C), enhancing insect epidermal penetration and diffusion in the tissues [40]. Of the three types of traditionally used chemical insecticides, dichlorvos, triazophos, malathion, beta-cypermethrin, cypermethrin, avermectin, imidacloprid, and their compound products are registered for production, sale and use against locusts in China [41]. Although phoxim and lambda-cyhalothrin are not registered on the Official China Pesticide Information Website, they have good efficiency and might be used to manage the grassland locusts in future. Among them, β-cypermethrin has been the most commonly used insecticide for controlling *O. asiaticus* in Inner Mongolia over the last few decades, and the spraying dosage used was dependent on the density of *O. asiaticus* [42]. However, *O. asiaticus* has been reported to become resistant to pyrethroids with increasing frequency and usage [43]. Dong et. al. (2015) investigated the susceptibility to β-cypermethrin of *O. asiaticus* populations obtained from multiple areas of the Inner Mongolian steppes, and their results illustrated that the susceptibility of *O. asiaticus* populations to β-cypermethrin has been continuously reduced, which they attributed to elevated enzyme activities and mRNA expression levels of CarE and GST [16]. Given the emergence of increased insecticide resistance in *O. asiaticus* to conventional insecticides in field application, the rotation of insecticides with different modes of action to prevent the development of insecticide resistance in conventional systems is strongly suggested. Our results also illustrated that the biogenic insecticides showed high LC_50_ values to *O. asiaticus* adults from all three regions, probably because biogenic insecticides have shortcomings such as the longer time taken to cause pest mortality, and hence a more unstable control effect [44]. In fact, external biotic or abiotic factors including temperature, humidity, density, and the height of vegetation also significantly affect the control efficacy of biogenic insecticides [45]. At present, the main biological control agents against *O. asiaticus* include fungal insecticides (such as: *Metarhizium* species, *Beauveria bassiana* Bals.), poxvirus, and natural enemies (such as birds and small predators). Among them, *M. anisopliae* has been proven to have the most effective control effect against *O. asiaticus*; it has the advantage of being green, safe, and has long duration in the field, but has a slow effect on outbreaking insects [46]. Chemical insecticides are fast-acting and highly effective, but their excessive use generates residues which pollute the environment and can also have a negative impact on food safety for humans and animals [47]. However, the use of a combination of these insecticides can effectively solve these challenges by working synergistically to achieve better pest control, and can also address the negative effects of excessive use of chemical agents [48].

Chemical and biological control are both important for the management of *M. palpalis* in the desert steppes of northern China [49]. Traditional insecticides such as phoxim, methomyl, λ-cyhalothrin, and β-cypermethrin have been used in these bio-systems for many years, but they may also lead to reductions in insect diversity and natural enemy populations. In the past decade, insecticides with novel and unique modes of action have shown high toxicity to insect populations while being relatively non-toxic to natural enemies [50]. Our results suggest that spinosad could prove to be essential to the integration of chemical and biological control in IPM programs, as it was the most toxic insecticide to *M. palpalis* under laboratory conditions. However, we also observed the low mortality of *O. asiaticus* after spinosad topical application, suggesting that the toxicity of spinosad may vary depending on the route of exposure for specific species. The toxicity of spinosad to *M. palpalis* was higher than that of pyrethroids, carbamates, and organophosphates, and it may also provide better selectivity to predators when compared with conventional insecticides if applied in the field [51]. Spinosad is more toxic by ingestion than by contact, so natural enemy insects that do not feed on treated plant tissues can therefore be protected from this insecticide [52], giving it the potential for use in the pest management in the northern steppes. In this study, broad-spectrum insecticides (i.e., λ-cyhalothrin, methomyl, and phoxim) were relatively highly toxic to *M. palpalis*, while biogenic insecticides (i.e., avermectin, azadirachtin, and matrine) were less toxic under laboratory conditions. Biogenic insecticides have, however, received extensive research attention and have broad prospects for development and application due to their positive characteristics, such as low toxicities, easy degradation, miscibility with other pesticides, and ability to control insects that have already developed resistance [53]. Avermectin, azadirachtin, and matrine have already been registered and applied. The results of this study indicate that spinosad is highly toxic and imidacloprid is moderately toxic to *M. palpalis* when compared to conventional insecticides, suggesting that both spinosad and imidacloprid would likely contribute to pest management in desert steppes and should be recommended. Spinosad and imidacloprid were selective with regard to acute toxicity, but further work is needed to evaluate their residual toxicity and their potential sublethal effects.

For the outbreaking leaf beetle, *G. daurica* in northern grassland, λ-cyhalothrin, β-cypermethrin, imidacloprid and phoxim were the most toxic insecticides when tested by a leaf-dip bioassay method. Among them, only β-cypermethrin and imidacloprid are officially registered insecticides for controlling leaf beetles. Exposure to imidacloprid may cause the reduced motility of *G. daurica*, probably because of the interaction of imidacloprid with acetylcholine receptors, resulting in a modification of receptor conformation [54]. There was an exception for spinosad when ingested, which caused high mortality. Our results can therefore be used as guidelines regarding which insecticides and spraying methods may be toxic to key insect species, and this information may assist pastoralists or famers during insecticide spraying times. For several biogenic insecticides, matrine, azadirachtin, and methoxyfenozide all showed low toxicity to *G. daurica* larvae from two regions, probably because biogenic insecticides require a long time to produce a toxic effect [44]. Among them, methoxyfenozide was reported as having low acute toxicity to *G. daurica*, when it was first applied in the desert steppes of northern China. Methoxyfenozide induces toxic symptoms in insects identical to those produced by other diacylhydrazine ecdysone agonists: it inhibits feeding, induces premature apolysis, causes abnormal cuticle deposition and other molting irregularities, and inhibits ecdysis in larval insects [55,56,57]. Therefore, the use of a combination of methoxyfenozide and other chemical insecticides can effectively achieve better pest control of *G. daurica* by producing a synergistic efficacy.

It is essential that insecticides with different modes of action are effective and long-lasting under field conditions while these grassland ecosystems are regenerating. The rational use of combinations of these chemical and biogenic insecticides may delay the development of insecticide resistance and prolong the time post-spraying that products are effective; an extended time of effectiveness would increase the possible exposure of insects to the active ingredients of insecticides, which are highly recommended for development as an important chemical control strategy for pest management in the desert steppes of northern China. Our data provide valuable information to develop the rational use of chemical and biogenic insecticides in a framework of integrated pest management in the desert steppes of northern China and to recommend some potential insecticide application strategies as follows: (i) officially registered insecticides are strongly recommended to control target key insects in desert steppes and grasslands. (ii) Pesticides of the same type with good efficiency can be used in rotation, such as: phoxim, methomyl, lambda-cyhalothrin etc. (iii) The use of combinations of chemical and biogenic insecticides with different modes of action can be adopted to expand the insecticidal spectrum and delay resistance. (iv) Insecticides with novel and unique modes of action should be investigated to evaluate their toxicity in labs and under field conditions. (v) Insecticide resistance levels should be monitored constantly. As this work was carried out under laboratory conditions, further studies will be carried out under field conditions to determine the best formulations of compounds which show promising efficacy.

## 4. Conclusions

The use of *Oedaleus asiaticus*, *Myrmeleotettix palpalis*, and *Galeruca daurica* in toxicity tests in the present study confirmed the insecticidal effects of various types of chemical and biogenic insecticides. The results of this study will complement information from previous similar studies and inform future studies related to the control of outbreaking insects, such as *O. asiaticus*, *M. palpalis*, and *G. daurica* in the desert steppes of northern China. This research also provided baseline data on the use of chemical and biogenic insecticides for application in desert steppes.

## 5. Materials and Methods

### 5.1. Insects

**Figure 1:**
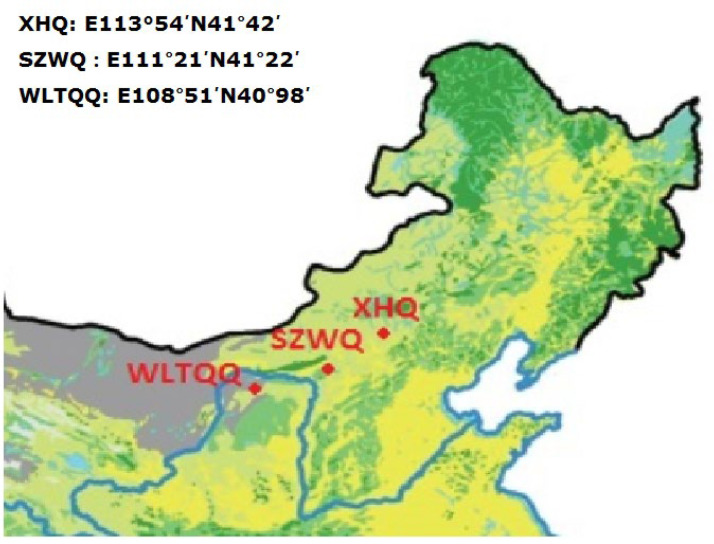
Location of the three sampling sites in desert grasslands of northern China used for evaluating toxicity in three outbreaking insects.

The field populations of three insect species, *O. asiaticus*, *M. palpalis*, and *G. daurica* used for insecticide toxicity evaluation, were collected from three locations in typical desert grasslands of northern China from 2021 to 2022. The outbreaks of the two locust species occur in late May and last until early July, while populations of the leaf bug *G. daurica* have high density during May. The collection sites are located in Xianghuang Qi (Xilinhot, Inner Mongolia, China. 40°98′ N, 113°54′ E, XHQ), Siziwang Banner (Agro-pastoral Field Experiment station, Ulanqab, Inner Mongolia, China. 41°22′ N, 111°21′ E, SZWQ), and Urat Qianqi (Bayannur, Inner Mongolia, China. 40°98′ N, 108°91′ E, WLTQQ) (Figure 1). The fifth-instar nymphs of *O. asiaticus* and *M. palpalis* were collected using sweep nets in late May and early June of 2021 and 2022 and immediately processed for bioassays.

### 5.2. Insecticides and Chemicals

The active insecticide ingredients phoxim (99%) were obtained from TianJin Pesticide Co., Ltd. (Tianjin, China); methomyl (90%) was from Jiangsu Fengshan Group Co., Ltd. (Yancheng, China); lambda-cyhalothrin (98%) and imidacloprid (95%) were purchased from Jiangsu Changlong Chemical Co., Ltd. (Changzhou, China); avermectin (91.2%) and beta-cypermethrin (95.2%) were gifted by HeBei VEYong Bio-chemical Co., Ltd. (Shijiazhuang, China) and Tianjin Longdeng Chemical Co., Ltd. (Tianjin, China), respectively. Methoxyfenozide was from Jiangsu Agricultural Academy. Technical grade 95% chlorantraniliprole was obtained from DuPont Agricultural Chemicals Ltd. (Shanghai, China).

The active biogenic insecticides azadirachtin (30%), and matrine (98%) were provided by Beijing Qingyuanbao Biological Technology Co., Ltd. (Beijing, China), and the analytical grade rotenone (96%) was brought online from SIGMA Co., Ltd. The other chemicals and solvents were purchased from commercial suppliers, including acetone, ethyl alcohol, ethyl acetate, and CO_2_ gas.

### 5.3. Bioassay

#### 5.3.1. Topical Application

The toxicity of each insecticide was determined by topical application. Groups of 30 fifth-instar nymphs were anaesthetized using *Drosophila* anesthesia equipment (YH-DACO2, Yi Hong technology company, Wuhan, China), and a 2.5 μL (*O. asiaticus*), 1 μL (*M. palpalis*) droplet of technical-grade insecticide in acetone was administered to the third abdominal segment using a semi-automatic dropper (PB-600 PAT, 3161323, Hamilton Company of America, Nevada, USA). Each insecticide was dissolved in acetone as a stock solution and serially diluted 5–8 times in a gradient, resulting in 10–90% mortality of the test insects. The control fifth-instar nymphs were treated with a 1% acetone solution. Each treatment was replicated three times with each repetition containing 10 fifth nymphs per plastic box (10 cm deep, 15 cm in diameter at top and bottom), meaning a total of 30 fifth-instar nymphs were used to test each of the insecticide or control, and all assays were performed at 22 °C ± 2 °C, RH 50% ± 10% and L 14 h: D 10 h. Nymphs that did not show any coordinated movement after probing with a soft brush were assumed to be dead after 48 h exposure.

#### 5.3.2. Leaf-Dip Bioassay

The toxicity of insecticides on *G. daurica* was determined using the leaf-dip bioassay method as described by Shelton (1993) with minor modifications. Stock solutions of each of the insecticides were prepared in 0.05% Triton X-100 sterile water to 5–8 dilutions, causing 10–90% mortality. The leek host plants, *Allium hookeri*, were planted in a greenhouse, grown without any pest-control chemicals or chemical fertilizers. Fresh leaves were cut into 2.5 cm diameter pieces and for each replicate, five leaf pieces were dipped in a solution of the diluted insecticide or control for 15 s, and allowed to air-dry at room temperature. The leaves were placed individually inside sterile dishes. The third-day second-instar larvae of *G. daurica* were selected and used for the bioassay. Each treatment was replicated three times with each replication containing 10 test bugs. The control larvae were treated with 0.05% Triton X-100 sterile water. All second-instar larvae were kept at 24 ± 2 °C, RH 40% ± 10%, and L 14 h: D 10 h, and mortality was recorded after 48 h exposure. The individuals were considered dead if they failed to move or twitch slightly when touched with a brush.

### 5.4. Data Analysis

Mortality data were corrected using Abbott’s formula (1987) [58] and analyzed by Probit analysis using POLO-Plus version 2.0 [59]. The relative toxic index is the ratio of LC_50_ values among different field populations.

## Figures and Tables

**Table 1:** Toxicity of chemical and biogenic insecticides against three field populations of *Oedaleus asiaticus* Bey-Bienko 2021–2022.

Insecticide	Chemical Structure	Year	Population	LC_50_ (95% FL) [ng a.i./Adult]	Slope ± SE	X^2^ (df ^a^)	RTI ^b^ (95% FL)
Phoxim	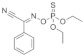	2021	SZWQ	301.58 [232.18–374.85]	2.50 ± 0.37	0.41 (3)	1
		2021	XHQ	369.96 [289.96–460.10]	2.42 ± 0.37	0.25 (3)	1.23
		2022	WLTQQ	281.90 [214.42–348.85]	2.89 ± 0.46	0.65 (3)	0.93
Methomyl	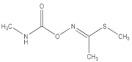	2021	SZWQ	940.13 [798.65–1102.83]	4.52 ± 0.82	0.74 (3)	1
		2021	XHQ	954.24 [783.35–1163.26]	3.26 ± 0.57	0.99 (3)	1.02
		2022	WLTQQ	906.55 [764.05–1061.96]	4.80 ± 0.92	0.46 (3)	0.96
Imidacloprid	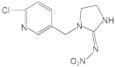	2021	SZWQ	292.89 [67.35–591.22]	0.97 ± 0.18	1.42 (3)	1
		2021	XHQ	717.91 [531.21–924.09]	2.03 ± 0.31	0.23 (5)	2.45
		2022	WLTQQ	462.59 [331.11–612.17]	1.80 ± 0.29	0.85 (3)	1.58
λ-cyhalothrin	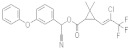	2021	SZWQ	334.90 [178.72–533.40]	0.99 ± 0.17	0.53 (4)	1
		2021	XHQ	878.04 [560.79–1281.50]	1.39 ± 0.26	0.48 (3)	2.62
		2022	WLTQQ	546.12 [317.84–788.50]	1.48 ± 0.28	0.66 (4)	1.63
β-cypermeth- rin	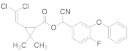	2021	SZWQ	213.48 [110.20–370.33]	1.06 ± 0.23	0.57 (4)	1
		2021	XHQ	286.34 [208.80–405.16]	2.10 ± 0.49	0.65 (4)	1.68
		2022	WLTQQ	174.95 [89.08–281.70]	1.37 ± 0.29	0.67 (4)	0.82
Chlorantrani- liprole	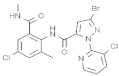	2021	SZWQ	1116.63 [375.05–4949.58]	1.01 ± 0.17	2.10 (4)	1
		2021	XHQ	1017.52 [605.26–1709.82]	1.04 ± 0.21	0.85 (4)	0.91
		2022	WLTQQ	435.50 [171.75–827.09]	1.04 ± 0.18	1.05 (4)	0.39
Avermectin ^c^	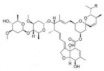	2021	SZWQ	11.99 μg [9.50–15.70]	1.99 ± 0.32	0.50 (4)	1
		2021	XHQ	15.09 μg [12.10–19.82]	2.34 ± 0.42	0.05 (4)	1.26
Spinosad	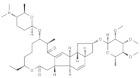	2021	SZWQ	488.06 [301.24–730.80]	1.44 ± 0.27	0.68 (4)	1
		2021	XHQ	571.48 [388.62–892.36]	1.20 ± 0.18	0.34 (5)	1.17
		2022	WLTQQ	578.99 [386.96–842.43]	1.54 ± 0.26	0.92 (4)	1.18
Matrine	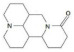	2022	SZWQ	1702.98 [455.41–2149.16]	3.16 ± 0.85	0.38 (3)	1
		2022	XHQ	1147.01 [748.61–1464.61]	2.73 ± 0.62	0.81 (3)	0.67
		2022	WLTQQ	1727.01 [1129.87–2221.80]	2.87 ± 0.80	0.34 (3)	1.01
Azadirachtin	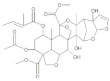	2022	SZWQ	1811.89 [1179.77–2393.49]	2.54 ± 0.75	0.51 (3)	1
		2022	XHQ	1198.20 [451.57–1820.36]	2.53 ± 0.52	1.10 (3)	0.66
		2022	WLTQQ	1069.17 [710.83–1412.23]	2.50 ± 0.63	0.46 (3)	0.59
Rotenone	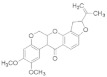	2022	SZWQ	750.63 [494.29–1015.93]	2.08 ± 0.44	0.98 (3)	1
		2022	XHQ	930.94 [552.91–1622.65]	1.17 ± 0.28	0.62 (3)	1.24
		2022	WLTQQ	737.61 [420.29–1028.81]	2.10 ± 0.52	0.73 (3)	0.98

^a^ Degree of freedom. ^b^ Relative toxic indexes. ^c^ Test time: 72 h.

**Table 2:** Toxicity of chemical and biogenic insecticides against two field populations of *Myrmeleotettix palpalis* 2021–2022.

Insecticide	Year	Population	LC_50_ (95% FL) [ng a.i./Adult]	Slope ± SE	X2(df ^a^)	RTI ^b^ (95% FL)
Phoxim	2021	SZWQ	216.72 [134.98–290.42]	2.03 ± 0.40	1.05 (6)	1
	2022	XHQ	247.89 [163.68–309.71]	3.67 ± 0.66	1.03 (4)	1.14
Methomyl	2021	SZWQ	200.95 [126.87–263.76]	3.30 ± 0.55	1.31 (5)	1
	2022	XHQ	164.64 [121.86–200.89]	2.88 ± 0.52	0.44 (4)	0.82
Imidacloprid	2021	SZWQ	101.94 [61.44–172.82]	1.10 ± 0.21	0.78 (3)	1
	2022	XHQ	109.99 [87.76–133.16]	3.39 ± 0.55	0.10 (3)	1.08
λ-cyhalothrin	2021	SZWQ	77.73 [55.00–98.52]	2.68 ± 0.50	0.21 (3)	1
	2022	XHQ	88.39 [66.06–114.89]	2.11 ± 0.32	0.22 (3)	1.14
β-cypermethrin	2021	SZWQ	279.73 [87.10–684.14]	1.44 ± 0.22	2.51 (4)	1
	2022	XHQ	215.54 [95.76–376.44]	1.59 ± 0.27	1.55 (4)	0.77
Chlorantraniliprole	2021	SZWQ	278.06 [118.76–447.50]	2.58 ± 0.48	1.30 (3)	1
	2022	XHQ	339.89 [257.88–424.45]	2.23 ± 0.31	0.83 (4)	1.22
Avermectin ^c^	2021	SZWQ	2.17 μg [1.33–3.16]	1.80 ± 0.27	1.28 (5)	1
	2022	XHQ	3.03 μg [2.35–3.84]	1.96 ± 0.34	0.35 (4)	1.40
Spinosad	2021	SZWQ	35.45 [23.65–48.43]	1.78 ± 0.32	0.28 (4)	1
	2022	XHQ	49.96 [32.71–70.96]	1.69 ± 0.33	0.10 (4)	1.41
Matrine	2021	SZWQ	526.71 [386.95–665.46]	2.37 ± 0.38	0.42 (5)	1
	2022	XHQ	749.73 [438.05–1249.35]	1.16 ± 0.30	1.09 (4)	1.42
Azadirachtin	2021	SZWQ	924.86 [720.98–1137.62]	2.78 ± 0.56	0.56 (3)	1
	2022	XHQ	662.80 [404.34–918.39]	2.09 ± 0.63	0.66 (3)	0.72
Rotenone	2022	SZWQ	337.50 [198.57–469.59]	1.85 ± 0.40	0.77 (4)	1
	2022	XHQ	198.20 [104.75–282.40]	1.92 ± 0.40	0.60 (5)	0.59

^a^ Degree of freedom. ^b^ Relative toxic indexes. ^c^ Test time: 72 h.

**Table 3:** Toxicity of chemical and biogenic insecticides against two field populations of *Galeruca daurica* 2021–2022.

Insecticide	Year	Population	LC50 (95% FL) [mg/L]	Slope ± SE	X2(df ^a^)	RTI ^b^ (95% FL)
Phoxim	2022	SZWQ	2.91 [1.53–4.74]	1.39 ± 0.27	0.20 (3)	1
	2022	XHQ	0.66 [0.26–1.24]	1.09 ± 0.23	0.72 (3)	0.26
Methomyl	2022	SZWQ	40.77 [27.30–52.25]	2.99 ± 0.80	0.54 (3)	1
	2022	XHQ	31.91 [22.55–39.91]	3.12 ± 0.69	0.79 (3)	0.78
Imidacloprid	2022	SZWQ	0.17 [0.10–0.31]	0.97 ± 0.15	0.59 (3)	1
	2022	XHQ	0.16 [0.07–0.32]	0.85 ± 0.17	0.28 (4)	6.82
λ-cyhalothrin	2022	SZWQ	0.73 [0.46–1.09]	1.69 ± 0.28	0.66 (3)	1
	2022	XHQ	0.56 [0.30–0.92]	1.24 ± 0.22	0.25 (3)	0.77
β-cypermethrin	2022	SZWQ	1.57 [0.91–2.61]	1.41 ± 0.27	0.55 (3)	1
	2022	XHQ	1.06 [0.55–1.86]	1.16 ± 0.24	0.58 (3)	0.68
Chlorantraniliprole	2021	SZWQ	56.12 [42.69–71.81]	2.03 ± 0.27	0.27 (4)	1
	2021	XHQ	38.17 [12.27–105.19]	1.40 ± 0.20	2.65 (4)	0.68
Methoxyfenozide ^c^ *	2021	SZWQ	3205.64 [2088.01–4319.27]	2.80 ± 0.59	1.12 (4)	1
	2021	XHQ	3802.80 [3008.44–4738.47]	3.53 ± 1.19	0.04 (3)	1.19
Spinosad	2022	SZWQ	50.10 [33.78–64.40]	3.00 ± 0.66	0.30 (3)	1
	2022	XHQ	28.12 [8.64–39.70]	2.46 ± 0.81	0.06 (2)	0.56
Matrine	2021	SZWQ	2267.18 [902.28–3977.09]	1.78 ± 0.35	1.11 (4)	1
	2021	XHQ	2269.82 [1384.18–3492.35]	1.45 ± 0.26	0.67 (4)	4.41
Azadirachtin	2021	SZWQ	3456.99 [1991.59–5242.97]	1.60 ± 0.42	0.20 (3)	1
	2021	XHQ	2982.66 [1788.12–4133.05]	1.91 ± 0.38	0.10 (4)	0.86
Rotenone	2021	SZWQ	751.73 [529.19–1050.16]	1.91 ± 0.38	0.82 (3)	1
	2021	XHQ	660.39 [350.54–1096.59]	2.17 ± 0.34	1.18 (3)	1.44

^a^ Degree of freedom. ^b^ Relative toxic indexes. ^c^ Test time: 240 h. * 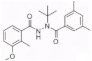.

## Data Availability

Data supporting reported results are available upon request.
